# Google trend analysis of climatic zone based Indian severe seasonal sensitive population

**DOI:** 10.1186/s12889-020-8399-0

**Published:** 2020-03-12

**Authors:** Jai Chand Patel, Pankaj Khurana, Yogendra Kumar Sharma, Bhuvnesh Kumar, Ragumani Sugadev

**Affiliations:** Defence Institute of Physiology and Allied Sciences, Defence Research and Development Organization, Lucknow Road, Timarpur, Delhi India

**Keywords:** Google trends, Seasonal sensitive population, Comorbid

## Abstract

**Background:**

Our earlier Google Trend (GT) Analytics study reported that the worldwide human population severely subject to four seasonal (sensitive) comorbid lifestyle diseases (SCLD) such as asthma, obesity, hypertension and fibrosis. The human population subject to seasonal variability in these four diseases activity referred as “severe seasonal sensitive population”. In India, the estimated burden of these four seasonal diseases is more than 350 million as on the year 2018. It is a growing crisis for India with a projected disease burden of 500 million in the year 2025. This study was aimed to decipher the genuine SCLD seasonal trends in the entire Indian population using GT and validate these trends in Indian climatic zones.

**Methods:**

GT is used to study the temporal trends in web search using weekly Relative Search Volume (RSV) for the period 2004 to 2017. The relative search volume (RSV) of the four-severe seasonal comorbid diseases namely Asthma, Hypertension, Obesity and Fibrosis were collected with and without obesity as the reference. The RSV were collected using the GT selection options as (i) Whole India (ii) Jammu and Kashmir (Cold zone) (iii) Rajasthan (Hot and Dry zone) (iii) West Bengal (Hot and Humid zone) and (iv) Uttar Pradesh state (Composite zone). The time series analysis was carried out to find seasonal patterns, comorbidity, trends and periodicity in the entire India and four of its states (zones).

**Results:**

Our analysis of entire India (2004–2017) revealed high significant seasonal patterns and comorbidity in all the four diseases of SCLD. The positive tau values indicated strong positive seasonal trends in the SCLD throughout the period (Table). The auto correlation analysis revealed that these diseases were subjected to 3, 4 and 6 months period seasonal variations. Similar seasonal patterns and trends were also observed in all the four Indian temperature zones. Overall study indicated that SCLD seasonal search patterns and trends are highly conserved in India even in drastic Indian climatic zones.

**Conclusions:**

The clinical outcome arise out of these observations could be of immense significance in handling the major chronic life style diseases asthma, hypertension, obesity and fibrosis. The possible strong comorbid relationship among asthma, hypertension, obesity and fibrosis may be useful to segregate Indian seasonal sensitive population. In disease activity-based chronotherapy, the search interest of segment of the population with access to Internet may be used as an indicator for public health sectors in the early detection of SCLD from a specific country or a region. As this disease population could be highly subject to the adverse effect of seasons in addition to life style and other environmental factors. Our study necessitates that these Indian populations need special attention from the Indian health care sectors.

## Background

The main environmental provocation from ambient climate, temperature change, elevated levels of air pollution impact on vulnerable individuals contribute to adverse change in their behavioural and physiological responses. In the historical era, the seasonal variation in non-communicable diseases was well recognized in ayurvedic and homeopathic medicines [1–3]. In the modern era, scant attention is paid on the seasonal perspective based critical examination of the non-communicable diseases due to human population across the globe has gradually extended with our ability (from heating to cooling) to achieve optimal habitat and work-place temperature control. In contrary, number of contemporary studies confirmed that enormous number of human populations across the globe influenced by the environment changes in particular to season variations with predominance winter peaks.

From the broader, beyond geographical location, the extent of seasonality in non-communicable diseases of a specific area is indeed attenuated or prevented by the micro climate of that region. There were several studies observed inconsistencies in both observe and report of seasonal variations in non-communicable diseases of a region-specific population. To reduce the interference of environmental provocations on genuine season effect, the locations studied should be geographically widespread where conventional data collection may be challenging and resource intensive. One tool Google Trends allows users to freely access three billion daily Google Search searches and provides data on widespread geospatial and temporal patterns in search volumes for user-specified terms [4–7].

Using Google Trend analytics, in seasonal perspective, our earlier study provided an indirect evidence of four comorbid seasonally sensitive diseases hypertension, asthma, fibrosis and obesity severely affect the human population worldwide in myriad of the above said environment together with ethnic variations named as “seasonal (sensitive) comorbid lifestyle diseases (SCLD)” [8]. The predicted seasonal comorbid association among asthma, hypertension and obesity is highly supported by clinical evidences [9–14]. Even though fibrotic diseases strongly associate with season, their seasonal comorbid association with obesity, hypertension, and asthma is poorly evaluated [15]. Our study predicted reverse in the comorbid seasonal search trends of SCLD between USA (Northern hemisphere) and New Zealand (Southern hemisphere).

Several clinical studies on Indian population reported many folds increase in the prevalence of the life style disorders [16–19]. For example the prevalence of obesity in India increased drastically to the alarming level of 30 to 40% in tune with the world population [20, 21]. The present study utilized GT from India to estimate the seasonality in these diseases especially SCLD and their comorbidity. In India, there are multiple factors could attenuate the seasonality in SCLD. The major factors are temperature and rainfall of the geographical locations, and the periods of searches (year, month and week) [22–27]. India has been divided into four major main climatic regions based on temperature and rainfall namely hot & dry, cold, composite and hot & humid [28]. Within diverse climatic regions, densely populated urban areas and distinct geographical features have potentially modulated both the overall climatic conditions and mean temperatures of those regions. In addition, in each climatic regions the Indian population exhibits diversities in social, culture, linguistics and in their genetic profiles [29, 30].

The present study tried to decipher the genuine seasonal effect in SCLD of whole Indian population with the following objective (i) to estimate the seasonal trends in SCLD and their comorbidity using GT for the period 2004–2017. The main outcome of the analysis includes: (a) highly significant seasonal search trends and comorbidity were noticed in SCLD. The similar significant seasonal trends were also validated in the four climatic zones of India. The significant seasonal search patterns in entire India and its climatic zones indicate that SCLD are growing crisis for India.

## Methods

### Selection criteria of Indian states for GT based on climatic zones

India is divided into 28 states on the basis of linguistics and culture [31]. In India, there are five climatic zones viz. cold, hot and dry, hot and humid, moderate and composite. Most of the Indian states belong to more than one climatic zone with few states lie in the single climatic zone. Indira et al., 2014 characterized and defined the boundary of the five climatic zones from the 15 years of per day weather reports from five weather stations (Srinagar, Jodhpur, Kolkata, Bangalore and New Delhi) belong to the five climatic zones [28]. The selection criteria of Indian states based on the characteristics of different climatic zones as follows (i) Cold climate zone with low solar radiation, in summer the maximum ambient temperature of 20–30 °C during the day and 0–10 °C at night, in winter the values are between 5 and 25 °C during the day and 0–10C at night, low relative humidity (25–40%) encompasses the Jammu and Kashmir state (ii) Hot & dry zone with high solar radiation 800-900 W.m2, in summer the maximum ambient temperature of 40–45 °C during the day and 20–30 °C at night, in winter the values are between 5 and 25 °C during the day and 0–10 °C at night, low relative humidity (25–40%) and low rainfall < 500 mm encompasses the Rajasthan state (excluding the eastern and southern fringes). (iii) Hot and humid climate zone with intense solar radiation, in summer the maximum ambient temperature of 30–35 °C during the day and 25–30 °C at night, in winter the values are between 25 and 30 °C during the day and 20–25 °C at night, low relative humidity (70–90%) encompasses the West Bengal State (iv) The Composite climate zone with high solar radiation in summer and low diffusion in monsoon, in summer the maximum ambient temperature of 10–25 °C during the day and 4–10 °C at night, low relative humidity (20–25%) in summer and reaches up to 55–95% in monsoon encompasses the Uttar Pradesh State (v) The moderate climate zone covering hilly areas and high plateau regions of India omitted from the analysis as it does not encompass any specific state as in the case of other climate zones.

### Google trend data collection

GT is used to study the temporal trends in web search using monthly and weekly Relative Search Volume (RSV). The relative search volume (RSV) of the four-severe seasonal comorbid diseases namely Asthma, Hypertension, Obesity and Fibrosis were collected with and without obesity as the reference. In the query, as a default option “all categories” and “all types of web search” were used. The RSV were collected using the GT selection options as (i) Whole India (ii) Jammu and Kashmir (Cold zone) (iii) Rajasthan (Hot and dry zone) (iii) West Bengal (Hot and humid zone) and (iv) Uttar Pradesh (Composite zone).

### Data analysis

Data processing and statistical analysis were carried out using ‘*trend’* and ‘*stats*’ packages in R version 3.5.0 [32, 33]. The Mann-Kendall and seasonal Mann-Kendall trend tests were used to detect overall trends significantly larger than the variance in the data for the SCLD search terms (α = 0.05). To determine the significant seasonal components, an exponential smoothing state space model with Box-Cox transformation, trend, and seasonal components (TBATS) were fitted to the data using ‘*forecast’* package [34]. Further, autocorrelation was performed to extract the cyclic patterns present in the data using *‘stats’*.

## Results

Worldwide, four life style disorders (hypertension, obesity, asthma and fibrosis) were recognized to have strong seasonal linkage. Furthermore, the complex comorbid connections among them demonstrated that such connections can be highly time varying public problem. No definite consensus currently exists to study the dynamic changes. Our earlier study revealed that such dynamic connections and change in their co-occurrence (comorbid) due to external stimuli (seasons) significantly associated with the time varying user internet search patterns [8]. To take this idea further, in our study we aim to test the hypothesis in a country population subject to moderate seasonal changes such as Indian population (average temperature varies from 25 °C to 45 °C). The climate of India comprises a wide range of weather conditions across a vast geographic scale and varied topography, making our generalisations problematic. In this context, we therefore proposed an evaluation of model scenarios, with temperature as variable. The temperature dependent data-driven model scenarios account for the four major climatic zones of India were individually analysed.

### Entire country weekly GT analysis without bench mark

**Fig. 1:**
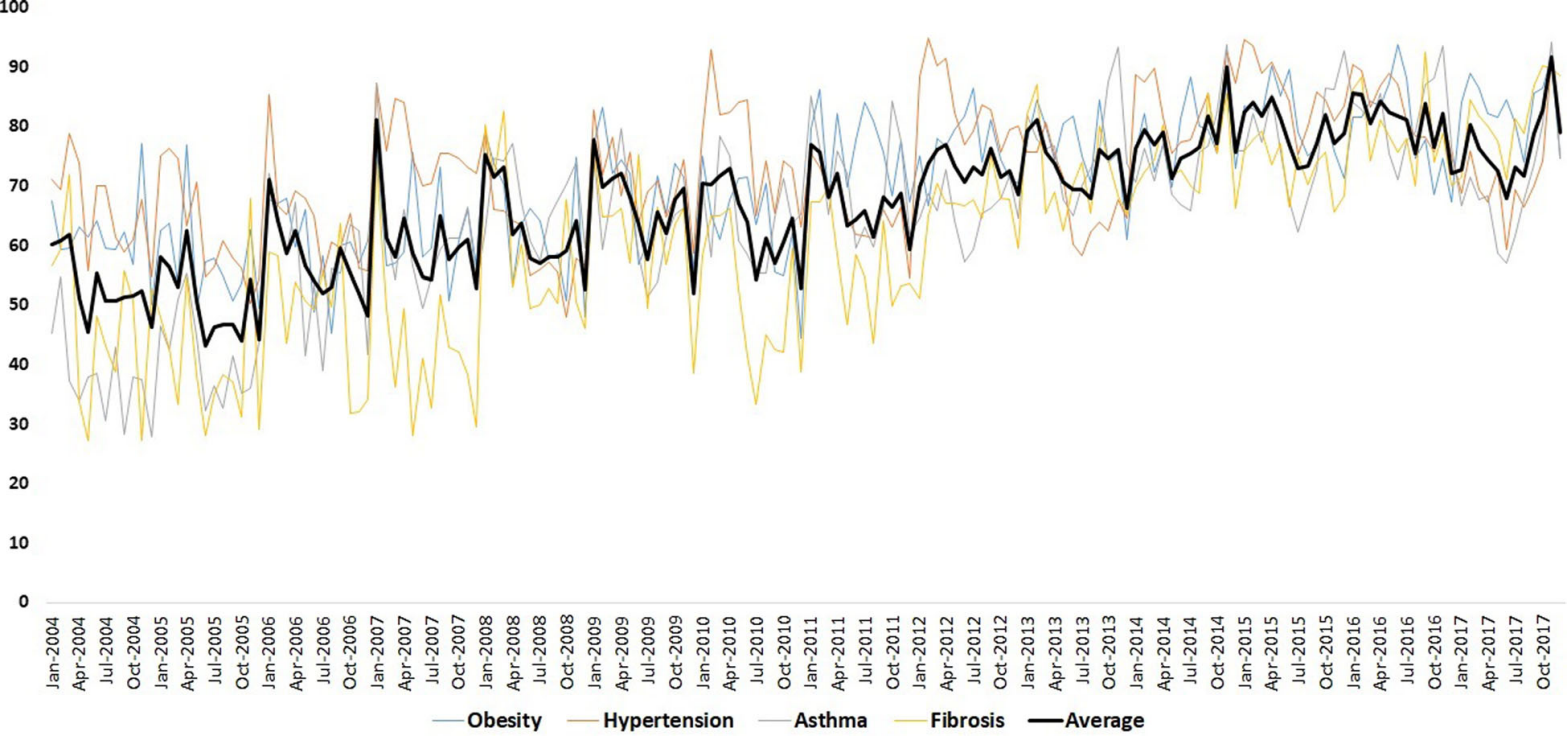
Entire India 4 weeks (monthly) moving average of weekly RSV (without benchmark) for SCLD from 2004 to 2017 were shown in four different colours. The overall average of the SCLD was shown in black colour (bold). Please note the positive (increasing) trends in the RSV from 2004 to 2017 with sharp hikes after the year 2010

**Fig. 2:**
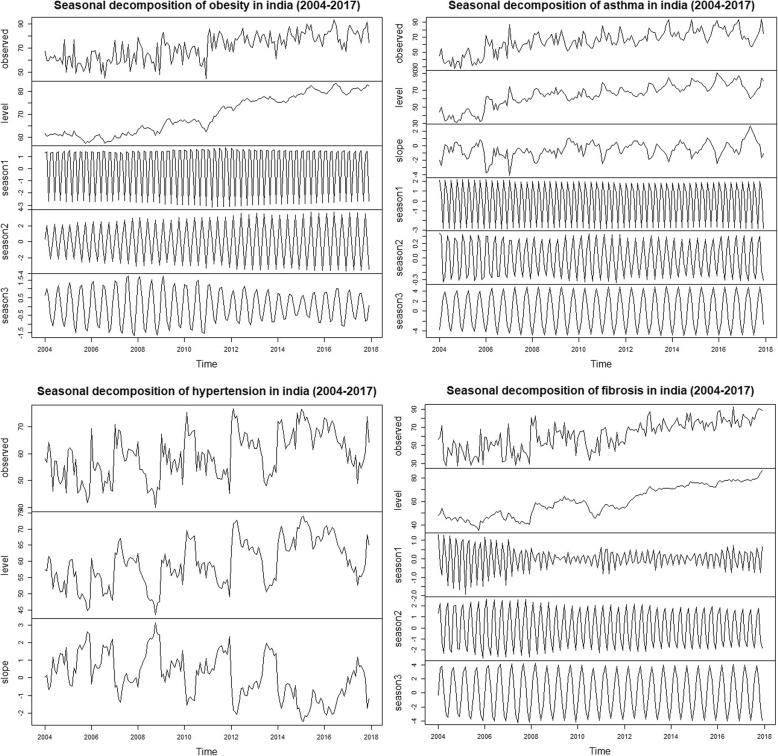
Seasonal and trend decomposition using TBATS for the SCLD for the weekly average (4 weeks) RSV from 2004 to 2017 without benchmark disease. Four weeks averaged weekly data were displayed for the SCLD in the top panels as observed (trend), level, slope and seasonal components 1, 2, and 3. Please note that strong seasonal patterns in all the SCLD except hypertension viz. 3 months (season 1), 4 months (season 2) and 6 months (season 3)

**Fig. 3:**
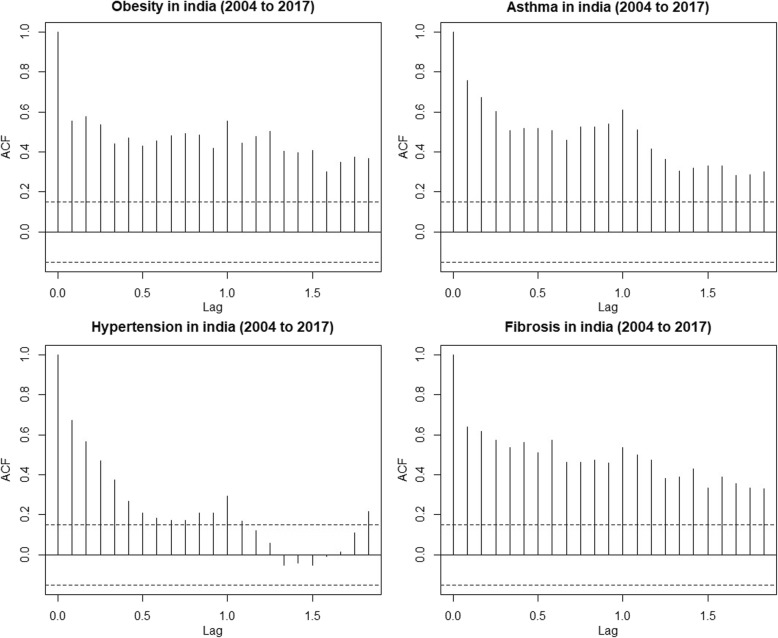
Autocorrelation of SCLD diseases for the 4 weeks averaged weekly RSV dataset from 2004 to 2017 without benchmark disease. Observed data were showing strong cyclic patterns in autocorrelation above significant line (dotted) except hypertension (weak) in the entire period

Without benchmark, the week wise and month wise varying RSV for the SCLD were analysed for seasonal trend in the period 2004 to 2017. The seasonal Mann-Kendall showed no seasonal trends in SCLD in both monthly as well as weekly datasets. The RSV plot showed high noise levels in the weekly GT data sets (Fig. 1). To reduce the noise levels, the weekly datasets were subjected to 4 weeks moving average to derive monthly datasets for each disease. Surprisingly, this approach improved the data quality considerably and resulted highly significant seasonal trends in all the four diseases of SCLD (Table 1). The positive tau values indicated strong positive trends search patterns in the SCLD. The RSV average of the four SCLD exposed the quantum jumps in the trends after the year 2010. Furthermore, the seasonal decomposition of the GT also revealed 4 and 6 months periodicity especially in asthma, obesity and fibrosis (Fig. 2). The autocorrelation analysis also revealed seasonal periodicity in the SCLD except hypertension (Fig. 3).

**Table 1:** Time series analysis of entire India 4 weeks (monthly) moving average of weekly RSV (without benchmark) for SCLD from 2004 to 2017

	Mean	Stdev	Seasonal Mann-Kendall				Mann-Kendall					Seasonal periodicity
			z	***p***-value	S	varS	z	S	varS	tau	***p***-value	
**Obesity**	70.77	11.15	10.24	< 0.01	649	3999	9.83	7174	531,445.3	0.51	< 0.01	3,4
**Asthma**	64.99	14.66	10.92	< 0.01	692	4002	10.19	7435	531,463	0.53	< 0.01	3,4,6
**Hypertension**	72.78	10.84	6.83	< 0.01	433	3999	5.84	4261	531,459	0.30	< 0.01	–
**Fibrosis**	61.04	16.18	11.43	< 0.01	724	4000	11.03	8044	531,460	0.57	< 0.01	4,6

*stdev* Standard deviation, *z* Mann-Kendall statistics, *S* (Positive differences- Negative differences), *varS* Variance of S

### Entire country monthly GT analysis with benchmark

**Fig. 4:**
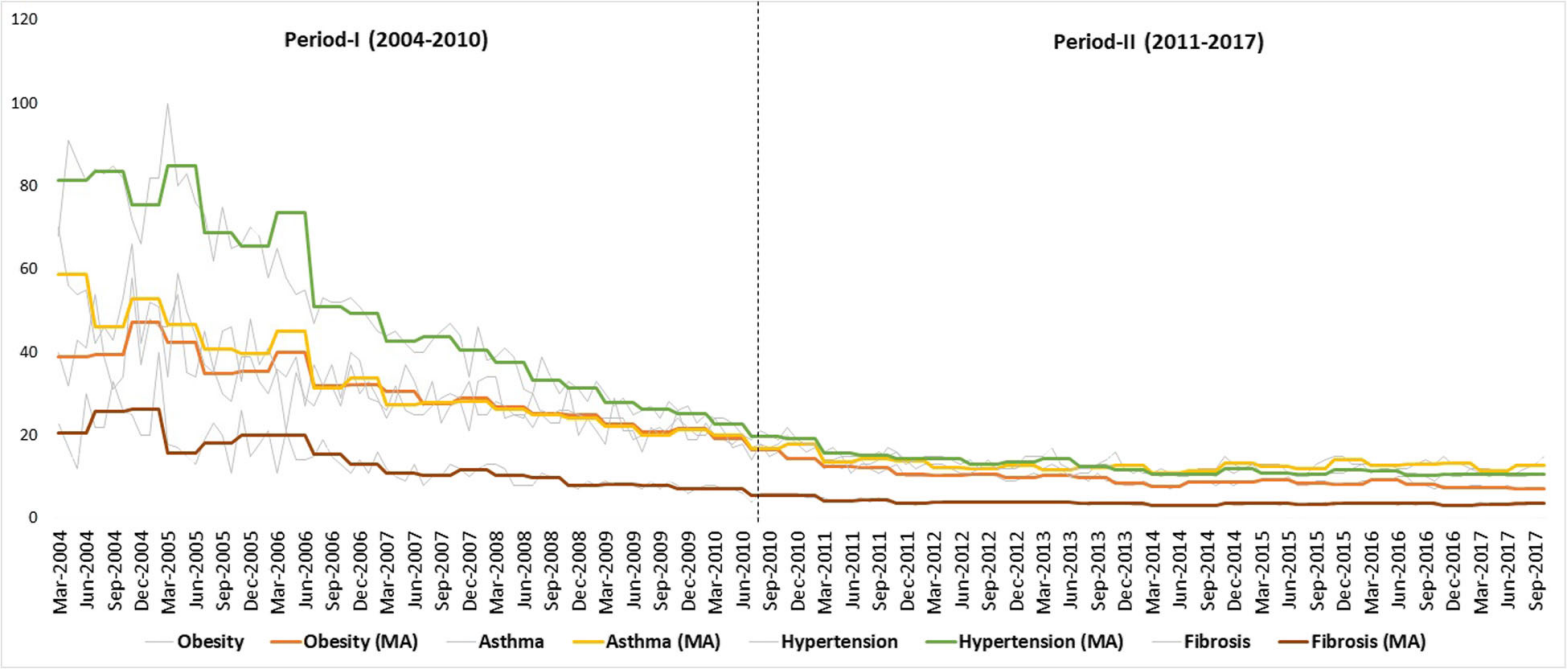
Entire India seasonal monthly RSV with benchmark were shown in thin grey lines and their corresponding seasonal moving average (4 months) from 2004 to 2017 were shown in four different colours labelled as MA in the brackets. Please note that sudden drop in the RSV with benchmark after 2010 was indicated to divide the period into I and II (dotted vertical line)

With benchmark, the monthly RSV from entire India was used to study the comorbid trends among SCLD. The seasonal Mann-Kendall captured significant seasonal comorbid trends in the four SCLD for the period 2004 to 2017. To reduce the noise levels and capture comorbid patterns, the monthly RSV of SCLD were subjected to seasonal moving average of window size 4. The window size was decided on the basis of GT weekly periodicity analysis (Table 2). All the four diseases seasonal comorbid patterns were well maintained in the entire period 2004 to 2017 (Fig. 4). Particularly, among SCLD the seasonal comorbid patterns between asthma and obesity were highly matched.

**Table 2:** Time series analysis of entire India seasonal (4 months) moving average of weekly RSV (with benchmark) for SCLD from 2004 to 2017

	Mean	Stdev	Seasonal Mann-Kendall			
			z	S	varS	***p***-value
**India**						
**Obesity**	19.63	12.36	−15.69	− 989	3961	< 0.001
**Asthma**	22.55	13.46	−13.35	− 839	3935	< 0.001
**Hypertension**	30.53	24.07	−15.80	−996	3962	< 0.001
**Fibrosis**	8.57	6.96	−14.15	− 876	3822	< 0.001

*stdev* Standard deviation, *z* Mann-Kendall statistics, *S* (Positive differences- Negative differences), *varS* Variance of S

### Zone wise GT analysis

Without benchmark, the zone wise weekly varying RSV of the SCLD were analysed for seasonal trend in the period 2004 to 2017. Before the analysis, the weekly datasets without benchmark were subjected to 4 weeks (monthly) moving average. The seasonal Mann-Kendall and Mann-Kendall captured statistically significant seasonal patterns and trends respectively in SCLD from different zones (Table 3). The zone wise seasonal trends of SCLD were highly comparable with that of entire India. The zone wise GT analysis with benchmark was omitted due to the weak RSV (Table 4).

**Table 3:** Time series analysis of zone wise 4 weeks (monthly) moving average of weekly RSV (without benchmark) for SCLD from 2004 to 2017

	Mean	Stdev	Mann-Kendall					Seasonal Mann-Kendall			
			z	S	varS	tau	***p***-value	z	S	varS	***p***-value
**Jammu Kashmir**											
**Obesity**	7.80	13.72	9.99	6137.00	377,519.70	0.58	< 0.01	9.87	522.00	2787.33	< 0.01
**Asthma**	7.72	13.49	10.10	6103.00	364,707.70	0.59	< 0.01	9.75	505.00	2674.33	< 0.01
**Hypertension**	13.52	20.34	12.39	8079.00	425,347.00	0.71	< 0.01	12.19	685.00	3149.67	< 0.01
**Fibrosis**	4.65	11.27	7.60	3794.00	248,961.30	0.46	< 0.01	7.51	320.00	1802.00	< 0.01
**Rajasthan**											
**Obesity**	22.71	18.92	10.44	7526.00	519,578.00	0.56	< 0.01	10.15	632.00	3868.00	< 0.01
**Asthma**	26.31	20.08	10.47	7584.00	524,639.30	0.56	< 0.01	10.20	640.00	3924.00	< 0.01
**Hypertension**	30.44	23.10	11.10	8056.00	526,928.00	0.59	< 0.01	11.27	705.00	3901.67	< 0.01
**Fibrosis**	13.91	15.62	7.76	5374.00	479,766.70	0.43	< 0.01	7.69	454.00	3471.33	< 0.01
**Uttar Pradesh**											
**Obesity**	27.22	23.35	10.95	7875.00	517,199.70	0.59	< 0.01	10.93	680.00	3858.00	< 0.01
**Asthma**	29.02	24.25	11.18	8060.00	519,598.70	0.60	< 0.01	10.81	675.00	3887.00	< 0.01
**Hypertension**	36.72	26.27	11.04	8019.00	527,681.00	0.58	< 0.01	11.10	700.00	3963.33	< 0.01
**Fibrosis**	19.716	17.80	9.42	6745.00	512,483.00	0.51	< 0.01	9.17	567.00	3808.33	< 0.01
**West Bengal**											
**Obesity**	26.64	22.68	11.63	8353.00	515,411.70	0.63	< 0.01	11.35	703.00	3825.00	< 0.01
**Asthma**	30.19	23.78	11.19	8070.00	520,314.70	0.60	< 0.01	10.88	679.00	3881.67	< 0.01
**Hypertension**	36.60	26.74	12.17	8845.00	528,004.30	0.64	< 0.01	11.79	742.00	3951.33	< 0.01
**Fibrosis**	20.08	18.67	9.57	6843.00	511,433.00	0.52	< 0.01	9.06	559.00	3796.33	< 0.01

*stdev* Standard deviation, *z* Mann-Kendall statistics, *S* (Positive differences- Negative differences), *varS* Variance of S

**Table 4:** Zone wise weekly RSV (with benchmark) for SCLD from 2004 to 2017

	Mean	Stdev
**Jammu Kashmir**		
**Obesity**	0.79	6.47
**Asthma**	0.08	0.33
**Hypertension**	0.11	0.39
**Fibrosis**	0.60	7.69
**Rajasthan**		
**Obesity**	4.73	9.86
**Asthma**	6.38	12.58
**Hypertension**	7.77	15.24
**Fibrosis**	3.06	8.16
**Uttar Pradesh**		
**Obesity**	4.67	7.65
**Asthma**	6.30	8.81
**Hypertension**	7.96	12.92
**Fibrosis**	3.25	6.73
**West Bengal**		
**Obesity**	3.87	8.23
**Asthma**	8.68	14.46
**Hypertension**	7.52	14.33
**Fibrosis**	2.76	7.91

*stdev* Standard deviation, *z* Mann-Kendall statistics, *S* (Positive differences- Negative differences), *varS* Variance of S

## Discussion

The present study successfully addressed all the objectives and revealed the following major outcomes.

### SCLD is a growing crisis for India

In India, according to I-Cube Internet user survey report 2018 more than 566 million have access to the Internet, accounting for 40% of the total population (https://imrbint.com/images/common/ICUBE%E2%84%A2_2019_Highlights.pdf). Such a large population of web users should provide reliable data for the SCLD surveillance in India. First time, our study revealed the highly significant seasonal patterns as well as more positive seasonal trends of SCLD in the internet search patterns from India. Furthermore, the internet weekly relative search volume (RSV) was also hiked after 2010 for SCLD. Similar positive trends were also noticed in the zone wise analysis. Several clinical studies of SCLD except fibrosis also reported the significant hike in the patient volumes after 2010 in India [25, 35, 36]. But clinical studies support our predicted seasonal comorbid trend among SCLD in overall Indian population was highly limited or negligible. Worryingly, the estimated diseases burden for the year 2017 was 207 million for hypertension, 135 for obesity, 37.9 for asthma and no registry for fibrosis to cross more than 500 million in the year 2022 [36–38]. Overall, our findings indicated that SCLD is a growing major crisis of health system in India. The seasonal severity and comorbidity could be addressed explicitly for the effective control and to take preventive measures of SCLD.

### Implication of GT in SCLD Chronotherapy

Chronotherapy is the synchronizing of drug concentrations to rhythms in disease activity, increasing efficacy as well as reducing adverse effects a major consideration to improve modern personalized medicine [39]. The rhythms in disease activity orchestrate either in terms of external clock time or internal circadian biological time [40, 41]. Clinical studies indicated that not only the external (to the local time) but also the internal circadian rhythms of human depend on season [42]. But the adverse effect of seasons on SCLD activity in the context of chronotherapy is underutilized and could be a major consideration to improve modern personalized medicine [43–47]. Our GT study also identified highly significant comorbid seasonal rhythm of periodicity of 3, 4 and 6 months in SCLD especially for asthma, obesity, fibrosis in the entire India. These seasonal rhythmic patterns could be readily exploited to estimate seasonality in SCLD to synchronize drug concentrations for better efficacy.

### Effect of temperature zones on SCLD

The zone wise GT data allowed us to validate the significant seasonal rhythmic patterns of SCLD in different temperature zones. Several clinical studies established significant relationship between seasonal change in life style diseases and temperature [48–53]. Seasonal trends from a wide range of weather conditions across a vast geographic scale and varied topography. For example, the seasonal changes in Indian states belong to northern, central, north-east and southern regions are highly distinct and drastic. Considering the fact, we studied the effect of different climatic zones on the SCLD seasonality to validate our results. Interestingly, as expected similar significant seasonal patterns and positive trends were noticed in all the four temperature zones. These results indicate that significant seasonal search patterns in SCLD is conserved in India even in the case of drastic climatic regions. Yet the conservation of comorbid seasonal search patterns among SCLD in different Indian climatic zones have to be established.

### Limitations

This study has several limitations in the text mining as well as electronic search (GT) that needs to be considered while interpreting the results. Most importantly, the individual performing the search is not necessarily suffering from the diseases. To validate our predictions, they should be correlated with clinical data. Meanwhile, the demographic characteristics were not available for the users who were performing the search. In addition, the seasonal patterns were not studied using any language other than English and with a search engine other than Google. Finally, the important caveat is only SCLD diseases terms used for GT analysis to study the effect on Indian population. In addition, the search behaviors of the individuals about the SCLD could not be assessed. According to the sources, around 74% of the internet user base will be comprised of internet users under 35 years old in 2016 [54].

## Conclusions

The clinical outcome arise out of these predictions could be immense significance in handling the major chronic life style diseases asthma, hypertension, obesity and fibrosis. The possible strong comorbid relationship among hypertension, obesity and fibrosis may be useful to classify Indian seasonal sensitive population. As this disease population could be highly subject to the adverse effect of seasons in addition to life style and other environmental factors. Our study necessitates that these population needs special attention from the Indian health care sectors.

## Data Availability

The datasets used and/or analysed during the current study are available from the corresponding author on reasonable request.

## Abbreviations

GT: Google trends
RSV: Relative search volume
SCLD: Seasonal sensitive comorbid lifestyle disease
